# Fecal microbiota transplantation in a rodent model of short bowel syndrome: A therapeutic approach?

**DOI:** 10.3389/fcimb.2023.1023441

**Published:** 2023-03-03

**Authors:** Salma Fourati, Anne Dumay, Maryline Roy, Alexandra Willemetz, Lara Ribeiro-Parenti, Aurélie Mauras, Camille Mayeur, Muriel Thomas, Nathalie Kapel, Francisca Joly, Maude Le Gall, André Bado, Johanne Le Beyec

**Affiliations:** ^1^ UMR-S1149, Centre de recherche sur l’inflammation, INSERM, Universite Paris Cite, Paris, France; ^2^ Sorbonne Université, AP-HP, Hôpital de la Pitié‐Salpêtrière‐Charles Foix, Service de Biochimie Endocrinienne et Oncologique, Paris, France; ^3^ Paris Center for Microbiome Medicine, Federation Hospitalo-Universitaire, Paris, France; ^4^ AP-HP, Hôpital Bichat -Claude Bernard, Service de chirurgie Générale OEsogastrique et Bariatrique, Paris, France; ^5^ UMR1319 - Micalis Institute, Institut National de Recherche pour l’Agriculture, l’alimentation et l’environnement (INRAE), AgroParisTech, Université Paris-Saclay, Jouy-en-Josas, France; ^6^ UMR-S 1139, INSERM, Universite Paris Cite, Paris, France; ^7^ AP-HP, Hôpital de la Pitié‐Salpêtrière‐Charles Foix, Service de Coprologie fonctionnelle, Paris, France; ^8^ Department of gastroenterology, IBD and nutrition Support, AP‐HP, CRMR MarDi, Hôpital Beaujon, Clichy, France

**Keywords:** short bowel syndrome, fecal microbiota transplantation, lactobiota, lactobacillaceae, hyperphagia, intestinal hyperplasia, short chain fatty acid

## Abstract

Extensive intestinal resection leads to Short Bowel Syndrome (SBS), the main cause of chronic intestinal failure. Colon preservation is crucial for spontaneous adaptation, to improve absorption and reduce parenteral nutrition dependence. Fecal microbiota transplantation (FMT), a promising approach in pathologies with dysbiosis as the one observed in SBS patients, was assessed in SBS rats with jejuno‐colonic anastomosis. The evolution of weight and food intake, the lenght of intestinal villi and crypts and the composition of fecal microbiota of Sham and SBS rats, transplanted or not with high fat diet rat microbiota, were analyzed. All SBS rats lost weight, increased their food intake and exhibited jejunal and colonic hyperplasia. Microbiota composition of SBS rats, transplanted or not, was largely enriched with *Lactobacillaceae*, and α‐ and β‐diversity were significantly different from Sham. The FMT altered microbiota composition and α‐ and β‐diversity in Sham but not SBS rats. FMT from high fat diet rats was successfully engrafted in Sham, but failed to take hold in SBS rats, probably because of the specific luminal environment in colon of SBS subjects favoring aero‐tolerant over anaerobic bacteria. Finally, the level of food intake in SBS rats was positively correlated with their *Lactobacillaceae* abundance. Microbiota transfer must be optimized and adapted to this specific SBS environment.

## Introduction

Short bowel syndrome (SBS), a rare disease resulting in adults from an extensive intestinal resection, is the main cause of intestinal failure. When the length of remaining small bowel is less than 150-200 cm, the intestinal surface is below the minimum needed for the absorption of macronutrients, micronutrients, water and electrolytes, leading to malabsorption and diarrhea. Intravenous supplementation is vital to replace the important losses and to provide these patients with the required nutrients and vitamins. Although parenteral nutrition (PN) significantly improves the outcome of the SBS patients (Joly et al., 2018), it is also a source of life-threatening complications (Winkler and Smith, 2014; Madnawat et al., 2020).

In the first years following intestinal resection, spontaneous adaptive mechanisms appear in SBS patients (Tappenden, 2014a; Le Beyec et al., 2020) improving intestinal absorption and reducing dependence on PN. Accordingly about 50% of the SBS patients are weaned off PN within 2 to 3 years of resection (Messing et al., 1999; Pironi et al., 2016). Intestinal epithelial hyperplasia (Joly et al., 2009) and increased secretion of gut hormones, such as GLP2, are two key modifications of this post-resection adaptation. GLP2, mainly secreted by ileal and colonic entero-endocrine cells, is known to exert a strong intestinal trophic effect (Jeppesen et al., 2000; Gillard et al., 2016) and GLP-2 analogs are currently used to treat SBS patients (Jeppesen et al., 2012; Jeppesen et al., 2018). Despite the efficiency of GLP-2 analogs in reducing PN dependence, this life-long treatment induces variable responses among SBS patients (Jeppesen et al., 2018) and remains very expensive. Moreover, the long-term effects are not well elucidated, especially their possible neoplastic risk (Thulesen et al., 2004; Armstrong et al., 2020).

Preserving colon and an oral/enteral nutrition are critical for developing adaptive mechanisms (Nordgaard et al., 1994; Jeppesen, 2000; Tappenden, 2014b; Lambe et al., 2019) and reducing the need for PN (Messing et al., 1999; Amiot et al., 2013). A major modification of the colonic microbiota composition and richness is observed in SBS patients with colon (Briet et al., 1995; Budinska et al., 2020) and their *Lactobacillus*-enriched microbiota is also called lactobiota (Mayeur et al., 2016; Piper et al., 2017). It has been demonstrated that the presence of a colonic microbiota in the context of intestinal failure, even dysbiotic, allows recovering part of the massive unabsorbed food energy, clearly showing the importance of maintaining colon in continuity of the digestive flow (Nordgaard et al., 1994; Briet et al., 1995). But the SBS lactobiota may also have some deleterious effects: poor growth in young SBS patients (Piper et al., 2017), prolonged PN dependence (Engstrand Lilja et al., 2015) or even development of a D-lactate encephalopathy (Mayeur et al., 2013). Fecal microbiota transplantation (FMT) could be an interesting alternative therapeutic to modify intestinal microbiota composition and to boost energy extraction and synthesis of many nutrients, vitamins and metabolites (Rinninella et al., 2019).

Intestinal environment, and therefore diet, influences the colonic microbiota composition. It is known that a high fat diet (HFD) shapes the microbiota which, although inducing some immune side effects and stress to the small intestine epithelium (Caesar et al., 2015; Tomas et al., 2016; Kawano et al., 2022), becomes more efficient in extracting calories from the diet (Bäckhed et al., 2004; Turnbaugh et al., 2006; Ridaura et al., 2013). In the present study, we took advantage of this known HFD microbiota caloric effect to test whether such a microbiota can be implanted in SBS rats and strengthen the energy recovery capacities of their microbiota. Therefore, we realized a FMT from HFD rats to our previously validated model of SBS rats (Gillard et al., 2016), as a first attempt to evaluate a FMT as a possible therapeutic strategy in SBS individuals.

## Method

### Animal studies

All experimental procedures were performed according to the European Community guidelines and were approved by the Paris Nord local ethics committee and the Ministry of Education and Research (Apafis #8290). Eight-week-old male Wistar rats (n=30) weighting 300–350g (Janvier Breeding Center, Le Genest St Isle, France) were housed in groups of four in an animal facility with a 12h light-dark cycle, at an optimal temperature of 22°C. During a 4-weeks acclimatization period, rats were allowed to access water and solid chow ad libitum (standard diet, Genestil^®^, Altromin –Diet breeding rats/mice Vacuum C1324).

### Fecal microbiota transplant preparation

Feces from Wistar male rats (23 weeks-old), who received a high fat diet for 16 weeks (HFD: 45% fat diet/Lard ssniff E15744-34), were collected in sterile containers, diluted 1/10 in a sterile solution of 10% and disrupted mechanically (Ultraturrax. Pro200, Pro Scientific Inc., Monroe, CT) with limited bubbling for homogenization to limit oxygenation within 2 hours (Reygner et al., 2020). This operation was repeated during three days leading to 3 inocula immediately frozen and stored at -80°C (an aliquot of each was kept for microbiota analysis).

### Surgical and experimental procedures

From the 5^th^ to the 3^rd^ preoperative day, all rats received daily a broad-spectrum antibiotic treatment (Vancomycin: 0.5g/L, Amoxicillin: 1g/L, Metronidazole: 0.5%, Gentamycin: 80mg/ml) by oral gavage to deplete the majority of endogenous microbiota. The 2 following days a mixture of antibiotics (Vancomycin 0.5g/l; metronidazole 1g/l; amoxicilin 1g/l; potassium clavulanate 0.125g/l) was added daily to the drinking water. Feces samples were collected before and after antibiotic treatment. Rats were randomly assigned to Sham or SBS surgery group and, after 12 hours of fasting, were anesthetized by inhalation with 2,5% isoflurane (Iso vet^®^1000mg/g). They underwent a resection of 80% of small bowel, including the ileum and the ileocecal valve and 20% of colon, i.e 25cm distal after the ligament of Treitz and 4 cm distal to the cecum, followed by jejuno-colonic anastomosis (SBS,n=18), or a transection at 25cm from the ligament of Treitz, and a jejuno-jejunal anastomosis (Sham,n=12) as described in (Gillard et al., 2016). To prevent postoperative pain and infections, rats received subcutaneously xylocaïne 1% (100μl/100g) (Astra, France) and Penicillin G (20,000 units/kg) (Panpharma, Luitre, France). Dehydration was prevented by an intraperitoneal injection of 10 ml Bionolyte G5 (Sodium chloride [0.4%], Glucose [5.5%], Potassium Chloride [0.2%]) (Baxter, Maurepas, France), with free access to water for the first postoperative 24h. Liquid diet (Nutrison, Nutricia, France) was available ad libitum 48h after surgery. Access to solid food was restored 48h after surgery.

Fecal inocula (2ml) from HFD rats were administered by oral gavage 3 and 7 days after the surgery to half of the rats of each group (Sham-FMT, n=6 and SBS-FMT, n=9).

Body weight and food intake were monitored daily (from day 1 and day 4 respectively), serum and feces were collected at D15 and D27 and the rats were sacrificed at D27.

### Fecal collection and microbiota analyses

Microbiota from feces collected at day 15 were analyzed, by high-throughput sequencing using an Illumina MiSeq sequencer. 16S rDNA paired-end amplicon reads were processed using the FROGS 3.2 pipeline (Find Rapidly OTU with Galaxy Solution) (Escudié et al., 2018). Bacterial composition and diversities were estimated using FROGSTAT phyloseq tools. Linear discriminant analysis (LDA) coupled with effect size (LEfSe) algorithm was applied to identify bacterial taxa differences between groups (Segata et al., 2011). Details in **Supplementary Data**.

### Morphological and biochemical analyses

An average of 10 crypts (colon/jejunum) and 10 villi were measured per rat in 5-micron-thick sections stained with hematoxylin phloxine saffron (HPS) using TRIBVN CaloPix software (TRIBVN, Chatillon France). Details in **Supplementary Data**. See **Supplementary Data** for biochemical analyses

### Statistical analyses

Statistical analyses were performed using GraphPad Prism version 9.1. (Graphpad software, San Diego, CA, USA). Correlations were determined with Pearson test when Normality was verified, or Spearman test for non-parametric variables. Bacterial abundance and alpha-diversity were compared by Mann-Whitney test, Kruskal-Wallis test or Wilcoxon test (significance level 0.01). The significance of beta-diversity metrics was assessed by PERMANOVA test (Permutational Multivariate Analysis of Variance Using Distance Matrices).

## Results

### Rats follow up, nutritional and metabolic parameters

Sham groups (with or without FMT) had a 100% survival rate. Two rats among the SBS groups (11.1%) died before the end of the follow up, one had peri-operative complications (SBS) and the second (SBS-FMT) was losing too much weight and had to be euthanized at day 24.

**Figure 1:**
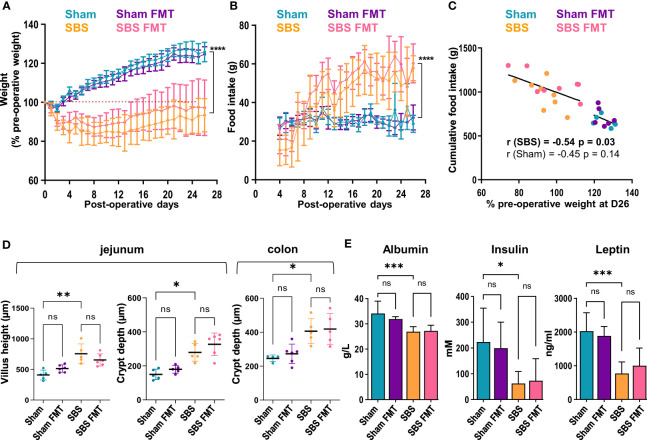
Weight follow-up and biological measurements in the 4 groups. **(A)** Weight expressed as percentage of the pre-operative weight of the Sham (blue, n=6), Sham-FMT (purple, n=6), SBS (orange, n=8) and SBS-FMT (pink, n=8) rats, during 26 days after surgery. **(B)** Food intake (in grams) of Sham (blue, n=6), Sham-FMT (purple, n=6), SBS (orange, n=8) and SBS-FMT (pink, n=8) rats, during 26 days after surgery. **(C)** Correlation diagrams between cumulative food intake and weight expressed as percentage of the pre-operative weight for all Sham rats (blue and purple, n=12) (Pearson test, r= - 0.45, ns: p> 0.05) and for all SBS rats (orange and pink n=16) (Pearson test, r= - 0.54, p < 0.0001) at day 26. **(D)** Comparaison of jejunal villus height and crypt depth, and colonic crypt depth in Sham (blue, n= 4 or 5), Sham-FMT (purple, n= 5 or 6), SBS (orange, n= 5) and SBS-FMT (pink, n= 5 or 6). **(E)** Albumin, insulin and leptin concentrations in Sham and SBS rats with or without fecal microbiota transplant, with n=6 for Sham, n=6 for Sham-FMT, n=8 for SBS and n=8 for SBS-FMT. Data are shown as mean ± SD, the asterisks indicate a significant difference between groups (Kruskal-Wallis test followed by Dunn’s *post hoc* test, *p<0.05, **p<0.01, ***p<0.001, ****p<0.0001, ns, p>0.05).

After the first two post-operative days, all rats had free access to food and Sham rats naturally gained weight throughout the follow-up with a mean of 125.9 ± 5% of their pre-operative at D26 (**Figure 1A**). On the contrary, SBS rats lost a mean of 14.7± 7% of their pre-operative weight during the first week (**Figure 1A**). Their weight stabilized at day 7 (85.3 ± 7%), then increased slowly until the end of the follow up to reach 93.4 ± 8/% of their initial weight at D26, and remained statistically different from Sham groups (p<0.0001). No differences in weight gain or loss were observed between rats with or without FMT throughout follow-up, whether they were in the Sham or SBS group (**Figure 1A**). Solid food was fully reintroduced on the third day and, as expected, Sham rats rapidly stabilized their food intake (FI) until the end of the follow-up (mean FI 30.8 ± 2g). In contrast, SBS rats increased their FI from day 7 (**Figure 1B**). The mean FI of SBS rats was significantly higher than that of Sham from day 10 (38 ± 11g vs 28.7 ± 4g respectively, p = 0.02), reaching a mean FI at day 26 of 57.8 ± 6g vs 29.0 ± 4g for Sham rats (p<0.0001). No differences in FI were observed between rats with or without FMT throughout follow-up, whether they were in the Sham or SBS group. Greater interindividual variability was observed in SBS rats for weight change and food intake level. At the end of the follow-up the weight of SBS rats, but not of Sham rats, was negatively correlated with the cumulative food intake (**Figure 1C**) (all SBS rats: r= -0.541, p=0.03 or all SBS and Sham rats: r= -0.843, p<0.0001).

We analyzed the intestinal morphological adaptation in SBS rats on jejunal and colonic histological sections. Jejunal villi length and crypt depth were increased in SBS compared to Sham rats (755.4 ± 160um vs 408.4 ± 80um p=0.002 and 279.4 ± 52um vs 150 ± 31um, p=0.04, respectively) (**Figure 1D**). The colonic crypts were deeper in SBS compared to Sham rats (406 ± 74um vs 246 ± 19um, p=0.03) (**Figure 1D**). No differences were observed between Sham and Sham-FMT groups or SBS and SBS-FMT groups.

Plasma concentrations of albumin, insulin, and leptin were significantly decreased in SBS compared to sham rats, but no difference was observed between rats with FMT and their counterparts without FMT (**Figure 1E**).

### Diversity and composition of fecal microbiota after antibiotic treatment and after intestinal resection

Fecal microbiota was analyzed by 16S rDNA gene sequencing to evaluate the impact of antibiotic treatment and intestinal resection on bacterial abundance and composition.

**Figure 2:**
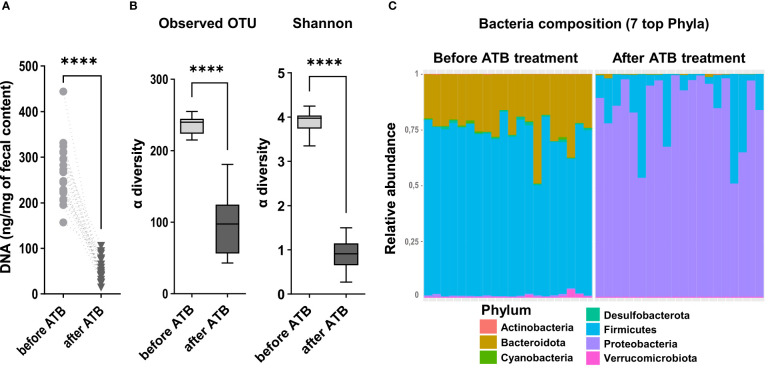
Quantification of the microbiota after antibiotic depletion and before intestinal resection. **(A)** DNA concentration (ng/mg of fecal content) in feces samples collected from all the rats included in the experiment, before (n=18) and after (n=18) the microbiota depletion by antibiotic treatment (ATB) (Wilcoxon test, ****p < 0.0001). **(B)** Alpha diversity of observed species in the microbiota of rats before and after the antibiotic treatment (ATB), estimated by observed OTUs counts and Shannon indices. Data are represented in boxes and minimum/maximum-ranges (whiskers) for n=18 rats before ATB and n=18 rats after ATB. Black lines indicate median values, (Wilcoxon test, ****p<0.0001). **(C)** Taxonomic composition and relative abundance of the 7 most abundant bacterial phyla per sample, determined by the relative proportion of 16S rRNA reads, before and after the microbiota depletion by antibiotic treatment (ATB). Each bar represents the bacteria composition observed in one rat.

We observed a significant decrease of DNA extracted from feces after antibiotic treatment, indicating that microbiota depletion was successful in all rats (**Figure 2A**). Accordingly, a large significant decrease in microbiota α-diversity was observed (observed mean OTU richness: 96.8 ± 40 vs 236.7 ± 12, p<000.1; Shannon = 0.9 ± 0.3 vs 3.9 ± 0.2, p<000.1; after vs before antibiotic treatment) (**Figure 2B**). The dominating phyla *Firmicutes* and *Bacteroidota* drastically declined after antibiotic treatment respectively by 80.1 ± 22% and 98.7 ± 2% (p<0.0001) (**Figure 2C**). Most of the remaining sequenced OTUs belonged to the *Proteobacteria*. We also observed during surgery that these antibiotics-treated rats had an enlargement of the size of their caecum compared to that of non-antibiotic-treated rats that we usually operate (personal observations). Altogether, these data and observations indicate that the antibiotic treatment was effective.

**Figure 3:**
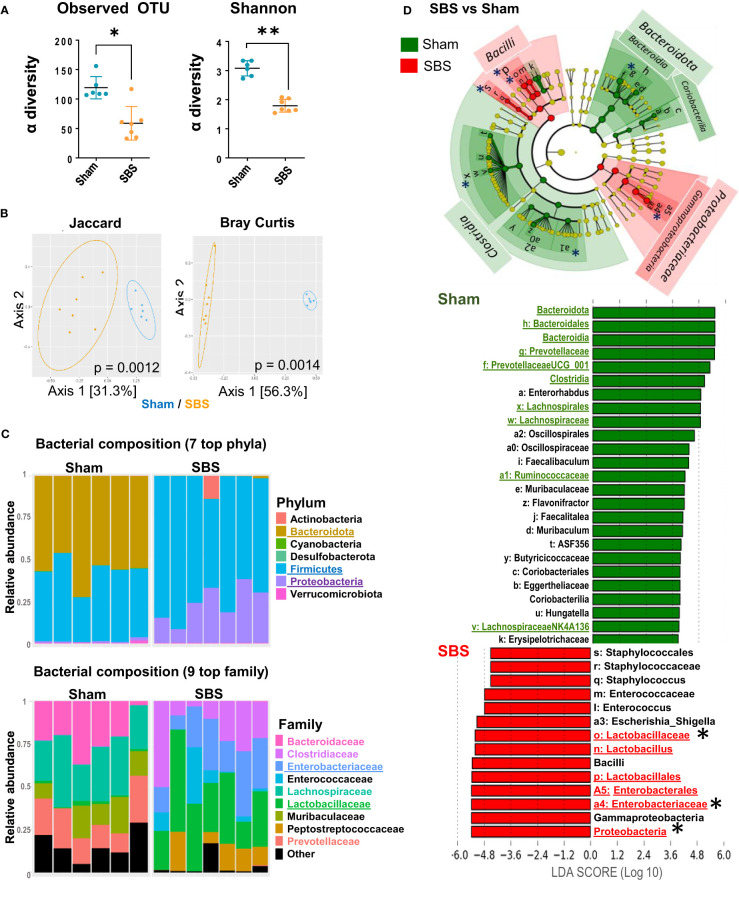
Fecal microbiota and metabolites composition in SBS rats compared to Sham. **(A)** Alpha diversity of observed species in the microbiota of SBS rats (orange, n=7) compared to Sham (blue, n=6), estimated by observed OTUs counts and Shannon indices (Data are represented as mean ± SD, and compared with Mann-Whitney test *p<0.05; **p<0.01). **(B)** Principal Coordinates Analyses (PCoA) of bacterial β-diversity in SBS (orange, n=7) and Sham (blue, n=6) rats, using Jaccard and Bray-Curtis distance plots. P-value were determined using PERMANOVA test (Permutational Multivariate Analysis of Variance Using Distance Matrices). **(C)** Taxonomic composition and relative abundance of the 7 most abundant bacterial phyla and the 9 top bacterial families per sample in SBS and Sham rats (determined by the relative proportion of 16S rRNA reads). Each bar represents the bacteria composition observed in one rat. **(D)** Linear discriminant analysis (LDA) integrated with effect size (LEfSe). Cladogram of differentially abundant taxa in the SBS rats (red, n=7) or Sham (green, n=6) rats. The LDA score indicates the effect size and ranking of each differentially abundant taxon, p<0.01 for LDA score >2.0. Bacterial groups of interest are underlined and highlighted with asterisk in the figure.

**Figure 4:**
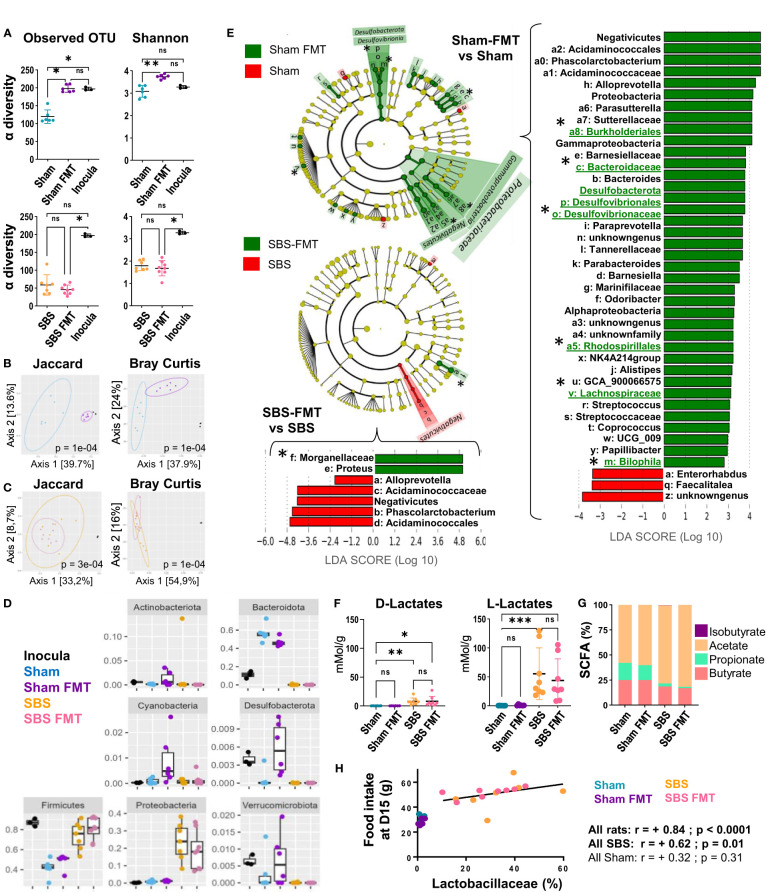
Analysis of the fecal microbiota bacterial composition and derived-metabolites, in the Sham and SBS rats with or without FMT. **(A)** Alpha diversity of observed species estimated by observed OTUs counts and Shannon indices in the microbiota of Sham (blue, n=6) and Sham-FMT (purple, n=6) or SBS (orange, n=7) and SBS-FMT (pink, n=8) rats and inocula (black, n=3); Data are represented as mean ± SD and significant differences between groups were determined using Kruskal-Wallis test and Dunn’s *post hoc* tests *p<0.05, **p<0.01, ns: p>0.05). **(B, C)** Principal coordinates analysis (PCoA) of bacterial β-diversity within Sham rats (blue, n=6), Sham-FMT rats (purple, n=6) and inocula (black, n=3) **(B)** and SBS rats (orange, n=7), SBS-FMT rats (pink, n=8) and inocula (black n=3) **(C)** using Jaccard and Bray-Curtis distance plots; P-value were determined using PERMANOVA test (Permutational Multivariate Analysis of Variance Using Distance Matrices). **(D)** Distribution and abundance of the 7 most abundant bacterial phyla among the 4 groups of rats (Sham (blue), Sham-FMT (purple), SBS (orange), SBS-FMT (pink) and in the inocula (black) determined by the relative proportion of 16S rRNA reads. Data are represented as mean ± SD and a Kruskal-Wallis test was used to compare groups. **(E)** Linear discriminant analysis (LDA) integrated with effect size (LEfSe). Cladogram of differentially abundant taxa in Sham-FMT (n=6) vs. Sham (n=6) and SBS-FMT (n=8) vs. SBS (n=7). The LDA score indicates the effect size and ranking of each differentially abundant taxon; p<0.01 for LDA score >2.0. Bacterial groups of interest are underlined and highlighted with asterisks in the figure **(F)** Lactate concentrations in the feces of Sham (blue, n=6), Sham-FMT (purple, n=6), SBS (orange, n=8), and SBS-FMT (pink, n=8) rats; (Kruskal-Wallis test followed by Dunn’s *post hoc* test, *p<0.05, **p<0.01, ***p<0.001, ns, p>0.05). **(G)** Proportions of the main SCFA (butyrate, propionate, acetate and isobutyrate) in the feces of Sham (n=6), Sham-FMT (n=6), SBS (n=8), and SBS-FMT (n=8) rats. **(H)** Correlation diagrams between food intake and the proportion of *Lactobacillaceae* in the fecal microbiota at day 15 for all Sham rats (blue and purple) (Spearman r =+0.32, p =0.310, n=12) or all SBS rats (orange and pink) (Spearman r= +0.70 p<0.01, n=15).

We analyzed the impact of intestinal resection on fecal microbiota (SBS vs Sham rats), at 15 days post-surgery. Based on the number of observed OTUs and Shannon indices, a loss of α-diversity was observed in SBS rats compared to Sham rats (**Figure 3A**). According to the β-diversity shown in **Figure 3B**, SBS and Sham rats exhibited a significant different composition of their microbial communities (p <0.0001). We then examined whether SBS rats developed a specific SBS microbiota enriched in *Lactobacilli. Firmicutes and Proteobacteria* represented the dominant phyla in the fecal microbiota from SBS rats (**Figure 3C**). Within the *Firmicutes*, *Lactobacillaceae* family was dominant in most SBS rats representing 15.7% to 59.8% of total bacteria (**Figure 3C** and **Supplementary Figure 1**). The *Proteobacteria* phylum was mainly composed of *Enterobacteriaceae*, which represented 8.1% to 38.1% of total bacteria in SBS rats (**Figure 3C** and **Supplementary Figure 1**). LEfSe analysis confirmed that the proportions of *Lactobacillaceae* and *Enterobacteriaceae* were highly increased in SBS compared to Sham rats (**Figure 3D**). The class of *Clostridia* was significantly lower in SBS rats, in particular with reduced *Lachnospiraceae* and *Ruminococcaceae*. The *Bacteroidota* were almost absent from the fecal microbiota of SBS rats while they represented about 50% of the Sham one (**Figures 3C**, **4D**). The *Prevotellaceae* group was also drastically reduced in SBS rats (**Figure 3C**).

We also measured lactate and SCFA concentrations to assess microbiota activity. L- and D-lactate fecal concentrations were significantly higher for SBS than for Sham rats (**Figure 4F**). The proportion of acetate increased in SBS compared to Sham rats (p=0.001), while butyrate (p=0.001) and propionate (p=0.01) decreased (**Figure 4G**).

Based on these results, SBS rat model exhibited a colonic microbiota with a low α-diversity dominated by *Lactobacillaceae* and *Enterobacteriaceae* and producing significantly more lactate than control rats. The analyses of SCFA composition showed an increase of acetate proportion at the expense of propionate and butyrate.

### Impact of FMT on bacterial biodiversity and taxonomic assessment

The FMT efficiency was assessed 7 days after the last gavage, i.e. 15 days post-surgery. The microbiota α-diversity of the inocula was higher than that of Sham or SBS rats (**Figure 4A**). FMT induced an enrichment of the microbiota diversity in Sham rats, as shown by the significant increase of microbiota α-diversity in Sham-FMT compared to Sham rats, but did not affect that of SBS-FMT rats (**Figure 4A**). The β-diversity (Jaccard and Bray-Curtis indices) highlighted significant dissimilarities between the microbiota composition of Sham and Sham-FMT rats (p<0.001) (**Figure 4B**). Furthermore, the Jaccard analysis showed that the composition of the Sham-FMT microbiota was closer to the inocula than the Sham rat microbiota. On the other hand, microbiota composition between SBS and SBS-FMT groups remained unchanged, as shown by clusters overlay, and were both clearly distinct from the inocula composition (**Figure 4C**).

Bacterial composition at the phyla level in the inocula and feces from the 4 rats’ groups are summarized in **Figure 4D**. In inocula, taxa belonging to *Firmicutes*, *Verrumicrobiota* and *Desulfobacterota* were significantly over-represented, and the latter two phyla were increased in Sham-FMT, but not in SBS-FMT, compared to Sham (**Figure 4D**). Altogether, these results indicate that FMT failed to take hold in SBS rats but that the microbiota from the inocula was, at least partially, engrafted in Sham rats.

Among the bacterial families that were significantly more abundant in Sham-FMT vs Sham on LEFSe analysis (with LDA score (log 10)>2, **Figure 4E**) we observed *Acidaminococcaceae, Desulfovibionaceae* including *Bilophila*, and *Bacteroidaceae* as well as other families belonging to the orders of *Rhodospirilalles* and *Burkholderiales* (**Figure 4E**). LEFSe analysis comparing Sham or Sham-FMT with inocula (**Supplementary Figures 2A, B**) showed that bacterial strains initially significantly more abundant in the inocula (vs Sham) were no longer differently represented between inocula and Sham-FMT. This included three of the bacterial groups previously identified as newly implanted (*Desulfovibionaceae, Rhodospirilalles and Bacteroidaceae)*, and also *Ruminococcus* (**Figure 4E** and **Supplementary Figures 2A, B**). None of the bacteria taxa implanted in Sham-FMT rats were increased in microbiota of SBS-FMT rats compared to that of SBS rats (**Figure 4E**), which is consistent with their similar α-and β-diversity indices (**Figures 4A, B**). The only overrepresented taxa in SBS-FMT compared to SBS belonged to the *Morganellaceae* family (order *Enterobacterales*) (**Figure 4D**).

FMT did not have an effect on the gut microbiota-derived metabolites analyzed. Neither the lactate concentrations nor the proportions of fecal SCFA (acetate, propionate and butyrate) were modified by FMT (**Figures 4F, G**).

Finally, as we observed heterogeneity in the nutritional phenotype of SBS rats (with or without FMT) we looked for relationships between key features and the predominant bacterial families in their microbiota as well as with their fecal metabolites. The proportion of *lactobacillaceae* measured at D15 was positively correlated with the food intake in SBS rats at that time (r=0.62, p=0.01) (**Figure 4H**), and almost correlated with their cumulative food intake calculated at D26 (r=+0.48; p=0.07 for SBS rats and r=-0.33 with p= 0.29 for Sham rats).

## Discussion

We validated for the first time in this Wistar rats SBS model with jejuno-colonic anastomosis, a microbiota modification with *lactobacillaceae* enrichment, increase in lactate production and modification of SCFA proportions, reflecting a lactobiota imprint (Gillard et al., 2017; Moens et al., 2019; Markowiak-Kopeć and Śliżewska, 2020). Despite the antibiotic pretreatment, the microbiota was that expected for SBS rats and confirmed that this SBS rat model recapitulates the changes in microbiota reported in SBS patients (Joly et al., 2010; Piper, 2018; Neelis et al., 2019; Budinska et al., 2020) or animal models of SBS (Lapthorne et al., 2013; Pereira-Fantini et al., 2016). This microbiota exhibited a low α-diversity compared to that of Sham rats, with a dominance of *Firmicutes*, mostly *Lactobacillaceae*, and an increased abundance of *Proteobacteria*, especially *Enterobacteriacea*. A significant reduction in obligate anaerobes belonging to *Bacteroidota* and *Clostridia* phyla, such as *Lachnospiraceae* and *Ruminococcaceae* was observed as well. The *Prevotellaceae* family was also depleted in SBS rats. These modifications are similar to those observed in the microbiota of SBS patients (Joly et al., 2010; Piper, 2018; Neelis et al., 2019; Budinska et al., 2020). Small bowel shortening certainly leads to acidic intestinal pH and higher oxygen concentrations in the colon, which probably explains the significant changes in the microbiota of SBS subjects (Joly et al., 2010; Mayeur et al., 2013; Mayeur et al., 2016; Budinska et al., 2020). Furthermore, as a disruption of the enterohepatic circulation of bile acids is known to impact microbiota composition (Boutte et al., 2021), its reduction in SBS subjects could also participate in their microbiota modification (Pereira-Fantini et al., 2016).

The α-diversities of the inocula were higher than those of Sham or SBS samples, probably because they were prepared from several obese HFD rats that had not been treated with antibiotics. Among the bacterial families successfully transplanted in the Sham-FMT microbiota, *Desulfovibionaceae* (i.e. *Bilophila and Rhodospirillales*) have been associated with weight gain or metabolic syndrome (Devkota et al., 2012; Clarke et al., 2013; Natividad et al., 2018). The inocula were enriched in *Firmicutes* over *Bacteroidota* which is also a known characteristic of obese subjects’ microbiota (Wang et al., 2016; Gomes et al., 2018). Since the rats were kept on a normal diet, it could partly explain why Sham-FMT rats did not gain more weight than Sham rats (Tomas et al., 2016). Furthermore, most studies reporting the effect of FMT from obese subjects on recipient weight have mainly been performed on germ-free rodents (Turnbaugh et al., 2006), not on microbiota-depleted rats (Ellekilde et al., 2014).

Remarkably, another common feature of the bacterial groups implanted in Sham-FMT rats was their anaerobe metabolism (Rey et al., 2010; Lu and Imlay, 2021) explaining the failure of these strains to resist and implant in the oxygen-enriched gut of SBS rats (Mayeur et al., 2016). In addition, the shorter transit times with accelerated digestive flows, together with higher intestinal concentrations of bile acids in SBS subjects, create a very challenging environment for bacterial colonization. From this perspective, the microbiota analysis at a single time point, i.e. one week after the FMT, may be considered as a limit of this experiment. A recent study reported that FMT on SBS piglets induced only transient changes to their intestinal microbiota that did not persist after 5 days (Hinchliffe et al., 2022). Furthermore, colonization by a “dysbiotic” microbiota from animals on HFD is transient if the diet is not maintained (Tomas et al., 2016). An increased proportion of *Morganellacea*e (belonging to the *Proteobacteria)* was observed in the SBS-FMT rats, but as they were not detected in the inocula, the hypothesis of an engraftment of this aero-tolerant family cannot be retained. An inflammatory environment in SBS intestine, exacerbated by FMT, may have promoted the growth of this bacterial group (Rizzatti et al., 2017).

Altogether, our results are encouraging for testing combinations of bacterial strains that would be more adapted to the luminal environment of SBS. A very recent study, carried out concurrently to our study, demonstrated that treatment of resected piglets, a model of pediatric SBS, with *Lactobacillus* and *Bifidobacterium* spp. increased both the diversity of their microbiota and their fecal SCFA concentrations (Pauline et al., 2022). Reinforcing *Firmicutes* at the expense of *Proteobacteria*, in particular by administering *lactobacillus* strains for which beneficial effects have been demonstrated, could increase diversity and SCFA, reduce possible pro-inflammatory bacterial imprint in SBS subject, limit muscle loss… (Ni et al., 2019; Liu et al., 2021; Giron et al., 2022; Pauline et al., 2022).

Beyond this FMT study, our analyses allowed us to demonstrate a positive correlation between food intake and *Lactobacillaceae* abundance, two characteristics of the SBS phenotype that were expressed with inter-individual variations. This observation is in agreement with other studies showing that *Lactobacillus* strains could modulate eating behavior (Sanchez et al., 2017; Saito et al., 2019). For example, administration of heat-killed *Lactobacillus brevis* to *in vitro* or *in vivo* models, induces increased secretion of the orexigenic hormone ghrelin, and increased appetite in an animal model (Saito et al., 2019).

The cumulative food intake in SBS rats was also inversely correlated with weight loss. This observation indicates that intestinal failure, due to resection, rapidly induced a significant weight loss (1st week) which stimulated food intake. In this case hyperphagia, together with jejunal hyperplasia, may have contributed to stabilization and recovery of their weight. The levels of food intake and weight change and the abundance of *Lactobacillaceae* were heterogenous in SBS rats, suggesting differences in adaptations between individuals. In SBS patients, who do not show important weight loss because it is prevented by PN, hyperphagia may also be observed (Cosnes et al., 1990; Crenn, 2004; Compher et al., 2009). It has been suggested that fat free mass may stimulate food intake in SBS patients, as well as in other populations (healthy or not) (Bétry et al., 2019). Interestingly strains from *Lactobacillaceae* family have also been shown to have beneficial effect on muscle mass (Liu et al., 2021; Giron et al., 2022). The relationships between the SBS microbiota and feeding behavior in SBS subject, the involvement of other organs, such as muscles (Wauters et al., 2023) and the mechanisms involved, will need to be further investigated in the future.

The FMT did not have an effect on the different SCFA proportions nor on the high lactate concentrations found in the feces of SBS rats. Nevertheless, as already reported in SBS animal model and patients (Gillard et al., 2017; Budinska et al., 2020), the large intestinal resection induced a major increase of lactate concentration, a modification of SCFA fecal proportions and a decrease of their fecal concentration, possibly due to the dilution caused by the diarrhea. Here, butyrate and propionate reductions are concomitant with lactate accumulation and with a microbiota composition change. This is likely due to the decrease in *Bacteroidota* (Salonen et al., 2014) and *Clostridia*, especially *Lachnospiraceae*, *Ruminococcaceae*, and *Negativicutes*, which are among the most important butyrate and/or propionate producer’s in the gut (Louis and Flint, 2009; Reichardt et al., 2014; Rivera-Chávez et al., 2016; Wang et al., 2020). The enrichment of the microbiota with butyrate producer strains would be interesting in SBS models considering the range of its beneficial actions exerted on intestinal cells (Roediger, 1982; Donohoe et al., 2011) and gut barrier functions (Martin-Gallausiaux et al., 2021), notably in SBS (Bartholome et al., 2004). However, most butyric acid-producing bacteria, such as *F. prausnitzii*, currently used as probiotic in gastrointestinal diseases (Miquel et al., 2013; Geirnaert et al., 2017), have an anaerobic metabolism and consequently are potentially unsuitable for SBS subjects. Future studies based on metagenomic analyses may help identify aerobic bacterial species possessing genes that encode enzymes for butyrate synthesis. The increase of acetate proportion in SBS feces can be explained by the decrease of butyrate and propionate proportions. Nevertheless, since acetate can be synthesized by most enteric bacteria as a fermentation product (Louis and Flint, 2017) it cannot be excluded that the intestinal SBS environment could also favor acetate-producing strains. Furthermore, acetate is also produced endogenously by intestinal epithelial cells, unlike butyrate and propionate (Moffett et al., 2020).

In this study, we demonstrated that FMT from HFD-induced obese rats used as donors to rats with large intestinal resection does not result in microbiota engraftment in SBS rats. The specific constraints of the new luminal gut environment shaped by the anatomical modification probably explain this lack of microbiota implantation. Further studies using FMT from well-adapted SBS rats or treatment with probiotics in this SBS rat model, mimicking adult SBS patients, will improve treatment options for this rare disease.

Extrapolation of these results to humans could be difficult. Indeed, in SBS patients there is a significant heterogeneity, due to the great variability of their associated pathologies, treatments and repeated exposure to antibiotics (Wang et al., 2017; Romani et al., 2020; Qiu et al., 2022). Yet, the presence of a lactobiota in SBS patients has been confirmed by several studies. Microbiota transfer, supported by an appropriate oral nutrition, is an interesting therapeutic option (Budinska et al., 2020; Boutte et al., 2021), that should be optimized using a microbial consortium tailored to the specific SBS luminal environment.

## Data availability statement

The datasets presented in this study can be found in online repositories. The names of the repository/repositories and accession number(s) can be found below: https://www.ncbi.nlm.nih.gov/, PRJNA856706.

## Ethics statement

The animal study was reviewed and approved by Paris Nord local ethics committee and the Ministry of Education and Research (Apafis #8290).

## Author contributions

JLB, AB, MLG, MT, FJ and NK; conceived and designed the study. SF, MR, AW, LRP, AM and CM performed the animal experiments and microbiota analyses. SF, MR and AD performed microbiota statistical analyses. SF, AD and JLB, interpreted the data. SF and JL wrote the manuscript. All authors contributed to the article and approved the submitted version.
