# Supplementary material for: Fecal microbiota transplantation in a rodent model of short bowel syndrome: A therapeutic approach?
Source: Front Cell Infect Microbiol. 2023 Mar 3;13:1023441. doi: 10.3389/fcimb.2023.1023441 (PMC10020656; doi:10.3389/fcimb.2023.1023441)
Supplement: Supplementary file 1 [file DataSheet_1.pdf]

Fecal microbiota transplantation in a rodent model of short bowel syndrome: a therapeutic approach?

Supplemental Methods

<u>Fecal collection and microbiota analysis:</u> Feces samples were collected fresh, flash frozen in liquid nitrogen and stored at -80°C. DNA was extracted from around 300 mg of fecal samples collected at day 15 using QIAamp Fast DNA Stool Mini extraction kit (Qiagen) and then quantified on a Qubit4 fluorimeter using the dsDNA HS Assay Kit (Life Technologies; USA). Microbiota composition and diversity were analyzed using an Illumina MiSeq sequencer, after a two-step PCR library preparation First, the V4 hyper-variable region of the 16S rDNA were PCR amplified (515-F: 5'GTGCCAGCMGCCGCGGT AA3', 806-R:5'GGACTACHVGGGTWTCTAAT 3'), and a second PCR was performed for samples indexing. The 16S rDNA paired-end amplicon reads were processed using the bioinformatics pipeline FROGS software (Find Rapidly OTU with Galaxy Solution), with the Galaxy platform (https://galaxy.migale.inra.fr/) (Escudié et al., 2018). Briefly, forward and reverse reads were filtered, trimmed for adaptor and PCR primers removal, merged, and chimeric sequences were removed. A total of 7,619,786 raw read pairs were generated, 7,089,043 were kept after quality filters and clustered in operational taxonomic units (OTUs). Taxonomic assignment was performed against the 16S SILVA version 138 pintail100 database. The species composition and diversities were estimated using the FROGSTAT phyloseq tools on Galaxy. Alpha-diversity within group was estimated using OTU richness (Observed) and Shannon diversity index. Beta-diversity between groups was evaluated by calculating Jaccard or Bray-Curtis distances between samples. Ordination using principal coordinate analysis (PcoA) was performed to represent biodiversity distribution at the OTU level between groups. Individual bacterial taxonomic differences between groups were determined by comparing abundance

values using the LEFSe algorithm (Linear discriminant analysis coupled with the effect size) (Segata et al., 2011).

Biochemical analyses: Plasma concentrations of albumin, insulin and leptin were measured by an automatic analyzer AU400 (Olympus Diagnostics, Rungis, France). Fecal SCFA (acetate, propionate, and butyrate) were measured using gas-liquid chromatography (Nelson 1020; Perkin-Elmer, St. Quentin en Yvelines, France), as described in (Lan et al., 2008) and expressed as percentage of the total SCFA extract. Fecal D- and L-Lactates were quantified using the Biosentec D/L lactic acid enzymatic kit (Biosentec, Toulouse, France), according to the protocol published by Mayeur and al. (Mayeur et al., 2013).

Morphological analyses : 5-micron-thick sections from Formalin-fixed and paraffin-embedded jejunal and colon samples were stained with hematoxylin phloxine saffron (HPS). Each slide was scanned then and analyzed, using respectively Aperio ScanScope CS System (Leica Microsystemes SAS, Nanterre France) and TRIBVN CaloPix software (TRIBVN, Chatillon France).

Escudié, F., Auer, L., Bernard, M., Mariadassou, M., Cauquil, L., Vidal, K., Maman, S., Hernandez-Raquet, G., Combes, S., Pascal, G., (2018). FROGS: Find, Rapidly, OTUs with Galaxy Solution. Bioinforma. Oxf. Engl. 34, 1287–1294. https://doi.org/10.1093/bioinformatics/btx791

Lan, A., Bruneau, A., Bensaada, M., Philippe, C., Bellaud, P., Rabot, S., Jan, G., (2008). Increased induction of apoptosis by Propionibacterium freudenreichii TL133 in colonic mucosal crypts of human microbiota-associated rats treated with 1,2-dimethylhydrazine. Br. J. Nutr. 100, 1251–1259. https://doi.org/10.1017/S0007114508978284

Mayeur, C., Gratadoux, J.-J., Bridonneau, C., Chegdani, F., Larroque, B., Kapel, N., Corcos, O., Thomas, M., Joly, F., (2013). Faecal D/L Lactate Ratio Is a Metabolic Signature of Microbiota Imbalance in Patients with Short Bowel Syndrome. PLoS ONE 8, e54335. https://doi.org/10.1371/journal.pone.0054335

Segata, N., Izard, J., Waldron, L., Gevers, D., Miropolsky, L., Garrett, W.S., Huttenhower, C., (2011). Metagenomic biomarker discovery and explanation. Genome Biol. 12, R60. https://doi.org/10.1186/gb-2011-12-6-r60

Supplemental figure 1: Comparison of the bacterial relative abundance of Lactobacillaceae and Enterobacteriaceae as percentage of the total bacteria in the fecal microbiota of the incocula (black, n=3), Sham (blue, n=6), Sham-FMT (purple, n=6), SBS (orange, n=7) and SBS-FMT (pink, n=8). The asterisks indicates the significant differences between groups, determined using Kruskal-Wallis test and Dunn's post hoc tests, **p<0.01.

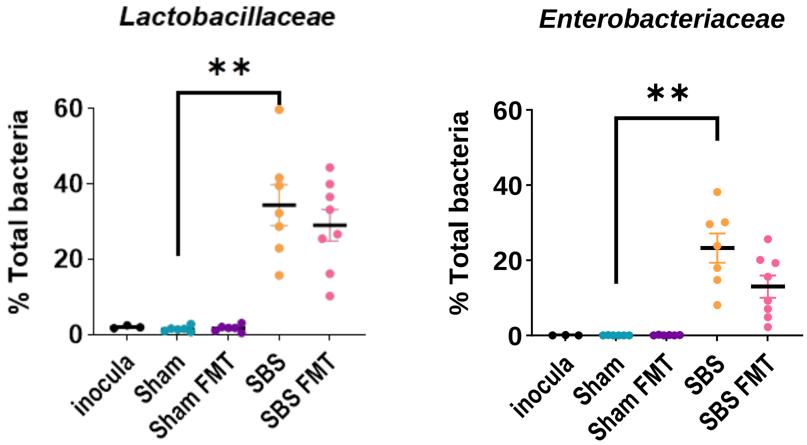

Supplemental figure 2: Linear discriminant analysis (LDA) integrated with effect size (LEfSe) ; **(A)** Cladogram of differentially abundant taxa in Sham (n=6) vs inocula (n=3); **(B)** Cladogram of differentially abundant taxa in Sham-FMT (n=6) vs. inocula (n=3) ; **(C)** Cladogram of differentially abundant taxa in SBS (n=7) vs. inocula (n=3) ; **(D)** Cladogram of differentially abundant taxa in SBS-FMT (n=8) vs. inocula (n=3). The LDA score indicates the effect size and ranking of each differentially abundant taxon; $p < 0.01$ for LDA score > 2.0.

A

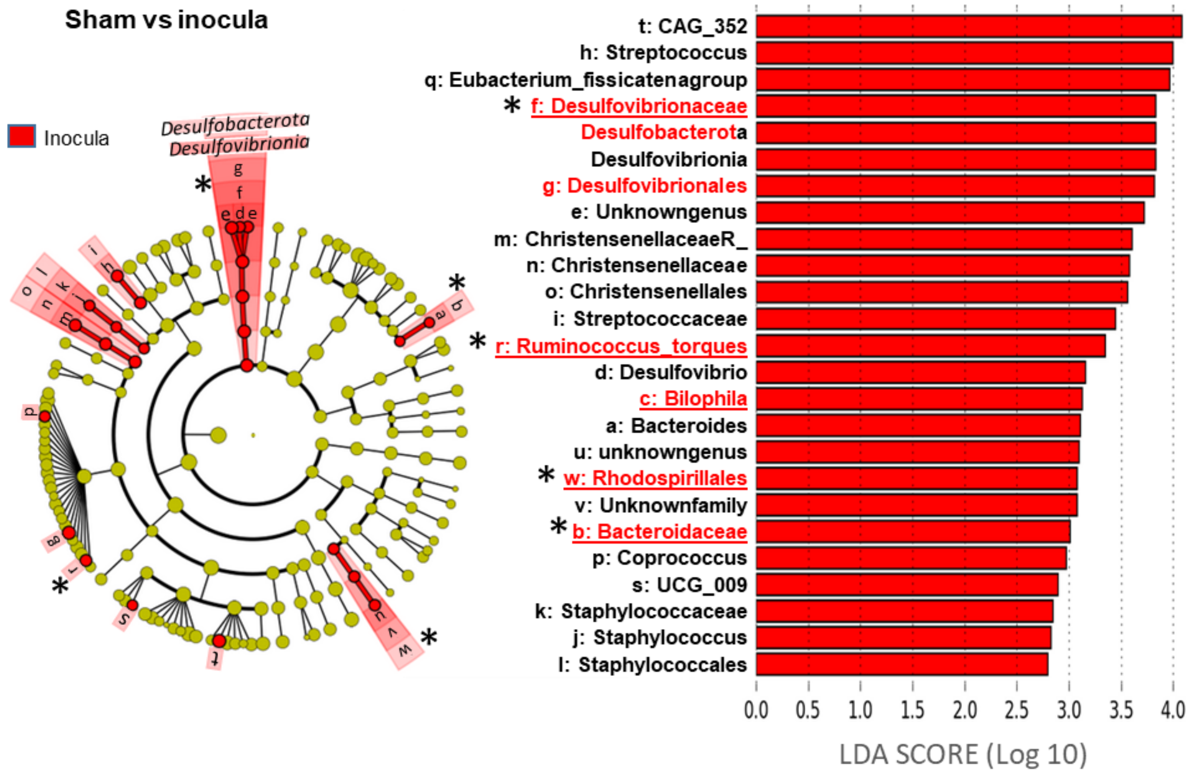

B

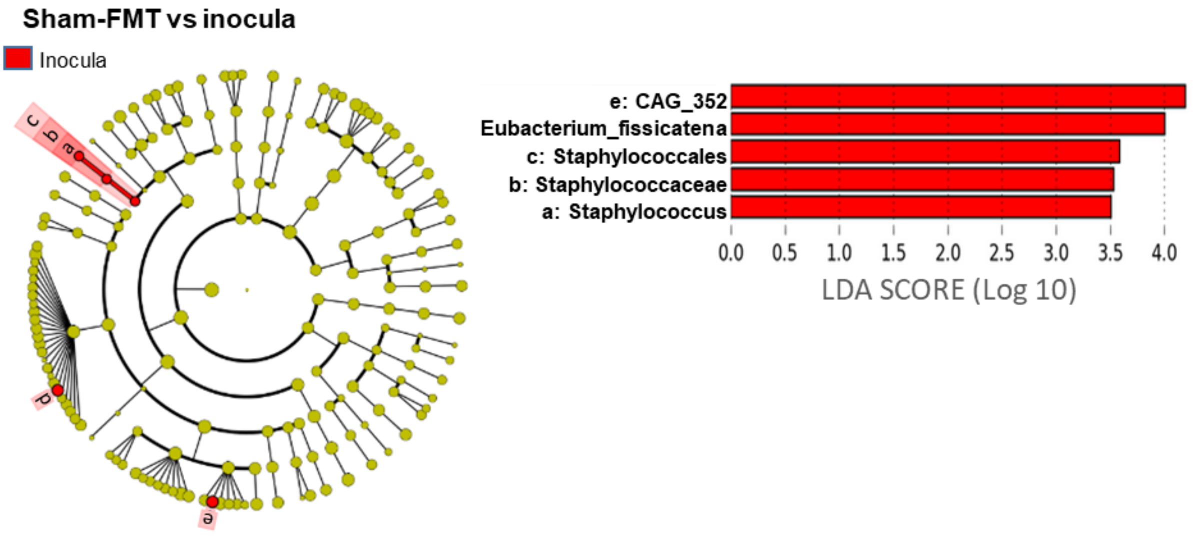

C

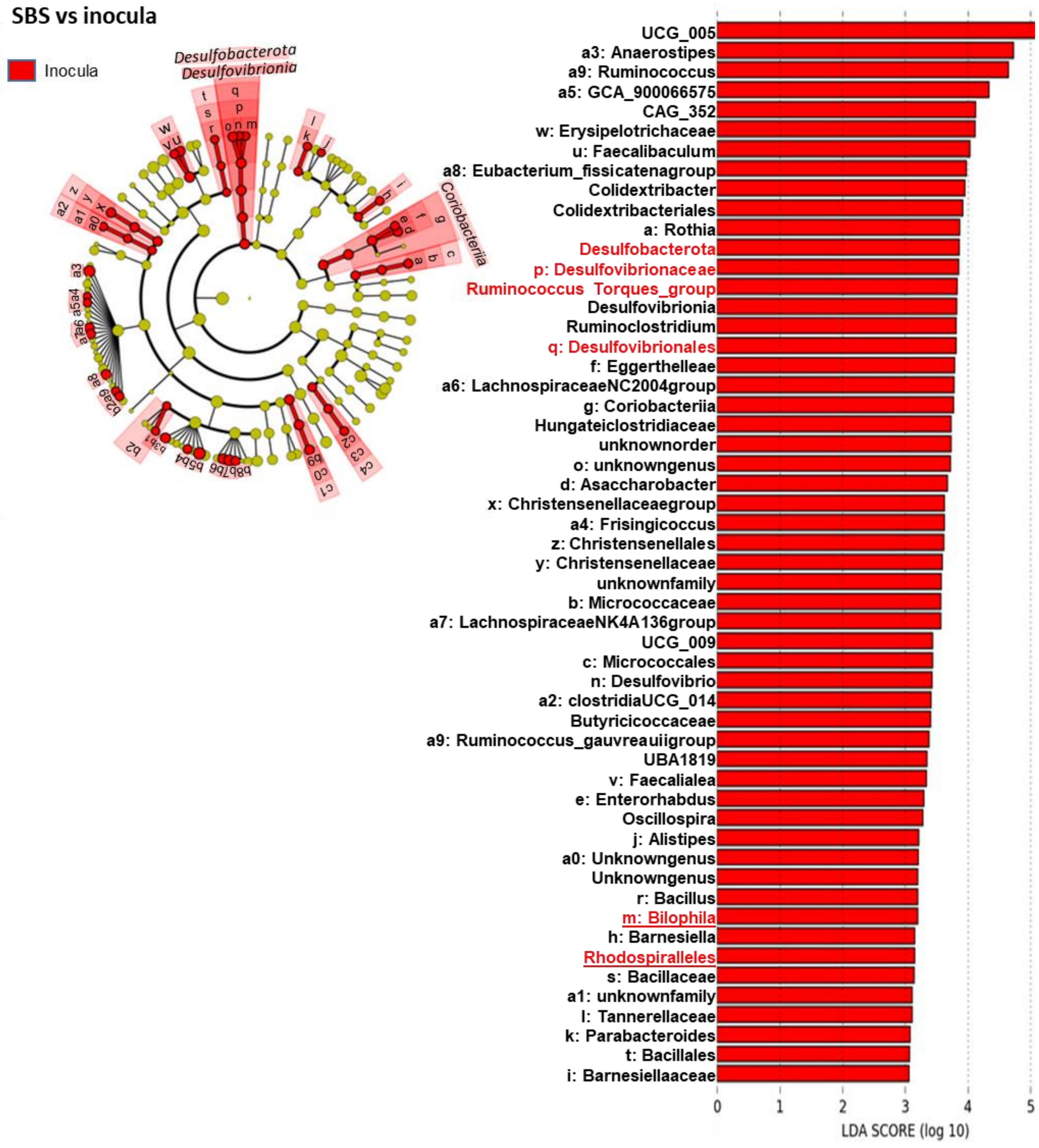
SBS vs inocula
Inocula
Desulfobacterota
Desulfovibrionia
Coriobacteriia
UCG_005
a3: Anaerostipes
a9: Ruminococcus
a5: GCA_900066575
CAG_352
w: Erysipelotrichaceae
u: Faecalibaculum
a8: Eubacterium_fissicatenagroup
Colidextribacter
Colidextribacteriales
a: Rothia
Desulfobacterota
p: Desulfovibrionaceae
Ruminococcus_Torques_group
Desulfovibrionia
Ruminoclostridium
q: Desulfovibrionales
f: Eggerthelleae
a6: LachnospiraceaeNC2004group
g: Coriobacteriia
Hungateiclostridiaceae
unknownorder
o: unknowngenus
d: Asaccharobacter
x: Christensenellaceaegroup
a4: Frisingicoccus
z: Christensenellales
y: Christensenellaceae
unknownfamily
b: Micrococcaceae
a7: LachnospiraceaeNK4A136group
UCG_009
c: Micrococcales
n: Desulfovibrio
a2: clostridiaUCG_014
Butyricicoccaceae
a9: Ruminococcus_gauvreauiigroup
UBA1819
v: Faecalialea
e: Enterorhabdus
Oscillospira
j: Alistipes
a0: Unknowngenus
Unknowngenus
r: Bacillus
m: Bilophila
h: Barnesiella
Rhodospiralleles
s: Bacillaceae
a1: unknownfamily
l: Tannerellaceae
k: Parabacteroides
t: Bacillales
i: Barnesiellaaceae
0
1
2
3
4
5
LDA SCORE (log 10)

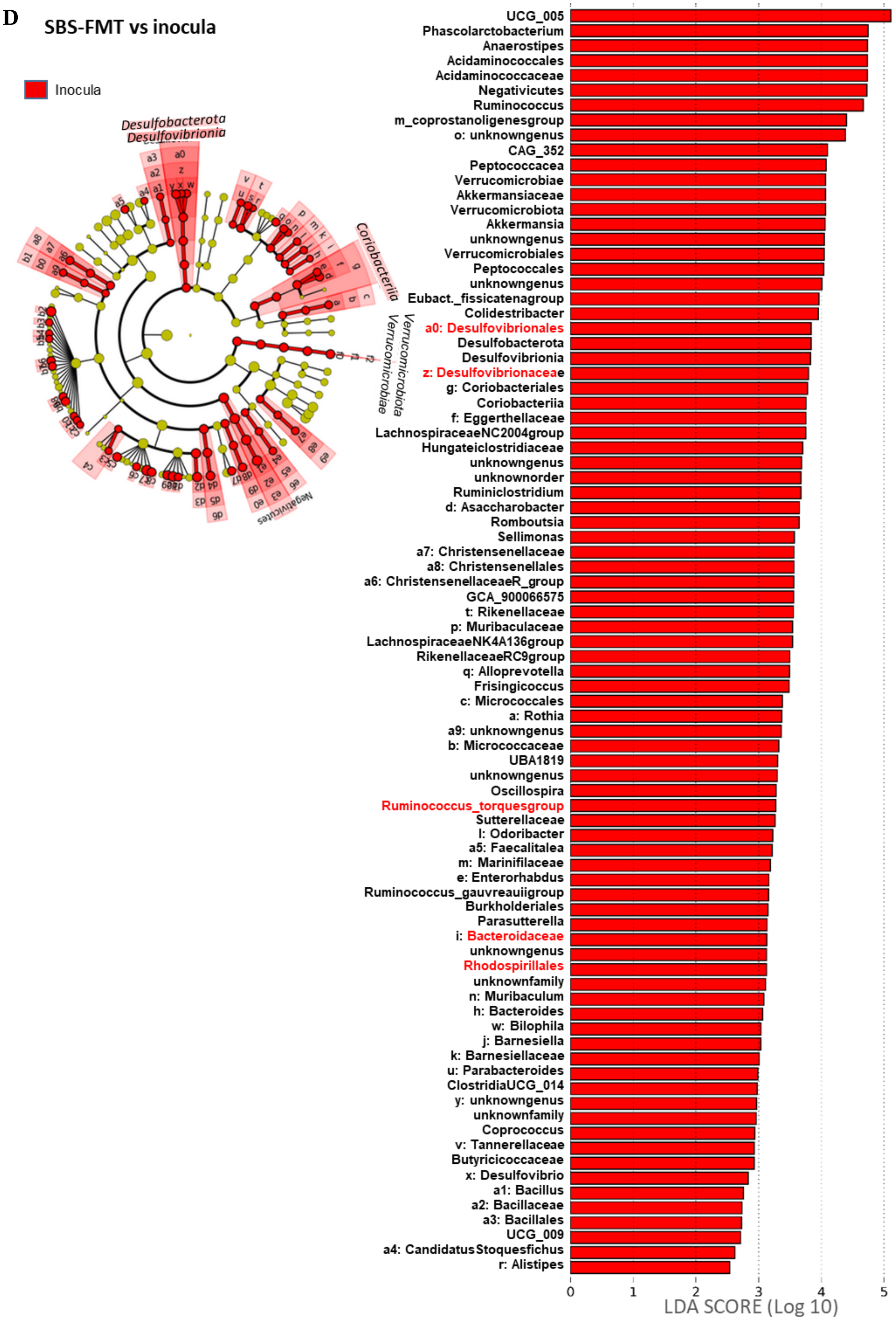
D
SBS-FMT vs inocula
Inocula
Desulfobacterota
Desulfovibrionia
Coriobacteriia
Verrucomicrobiota
Verrucomicrobiae
Negativicutes
UCG_005
Phascolarctobacterium
Anaerostipes
Acidaminococcales
Acidaminococcaceae
Negativicutes
Ruminococcus
m_coprostanoligenesgroup
o: unknowngenus
CAG_352
Peptococcacea
Verrucomicrobiae
Akkermansiaceae
Verrucomicrobiota
Akkermansia
unknowngenus
Verrucomicrobiales
Peptococcales
unknowngenus
Eubact._fissicatenagroup
Colidestribacter
a0: Desulfovibrionales
Desulfobacterota
Desulfovibrionia
z: Desulfovibrionaceae
g: Coriobacteriales
Coriobacteriia
f: Eggerthellaceae
LachnospiraceaeNC2004group
Hungateiclostridiaceae
unknowngenus
unknownorder
Ruminiclostridium
d: Asaccharobacter
Romboutsia
Sellimonas
a7: Christensenellaceae
a8: Christensenellales
a6: ChristensenellaceaeR_group
GCA_900066575
t: Rikenellaceae
p: Muribaculaceae
LachnospiraceaeNK4A136group
RikenellaceaeRC9group
q: Alloprevotella
Frisingicoccus
c: Micrococcales
a: Rothia
a9: unknowngenus
b: Micrococcaceae
UBA1819
unknowngenus
Oscillospira
Ruminococcus_torquesgroup
Sutterellaceae
l: Odoribacter
a5: Faecalitalea
m: Marinifilaceae
e: Enterorhabdus
Ruminococcus_gauvreauiigroup
Burkholderiales
Parasutterella
i: Bacteroidaceae
unknowngenus
Rhodospirillales
unknownfamily
n: Muribaculum
h: Bacteroides
w: Bilophila
j: Barnesiella
k: Barnesiellaceae
u: Parabacteroides
ClostridiaUCG_014
y: unknowngenus
unknownfamily
Coprococcus
v: Tannerellaceae
Butyricicoccaceae
x: Desulfovibrio
a1: Bacillus
a2: Bacillaceae
a3: Bacillales
UCG_009
a4: CandidatusStoquesfichus
r: Alistipes
0
1
2
3
4
5
LDA SCORE (Log 10)
